# Effect of Copper in Gas-Shielded Solid Wire on Microstructural Evolution and Cryogenic Toughness of X80 Pipeline Steel Welds

**DOI:** 10.3390/ma18153519

**Published:** 2025-07-27

**Authors:** Leng Peng, Rui Hong, Qi-Lin Ma, Neng-Sheng Liu, Shu-Biao Yin, Shu-Jun Jia

**Affiliations:** 1 Metallurgy and Energy Engineering, Kunming University of Science and Technology, Kunming 650000, China; lengpeng4782@126.com (L.P.); 15105633440@163.com (R.H.); liunengshengms@163.com (N.-S.L.); 2 Engineering Steel Institute, Central Iron and Steel Research Institute, Beijing 100081, China; maqilin0812@163.com

**Keywords:** gas-shielded solid wire, copper alloying, acicular ferrite, critical crack size, crack propagation angle

## Abstract

This study systematically evaluates the influence of copper (Cu) addition in gas-shielded solid wires on the microstructure and cryogenic toughness of X80 pipeline steel welds. Welds were fabricated using solid wires with varying Cu contents (0.13–0.34 wt.%) under identical gas metal arc welding (GMAW) parameters. The mechanical capacities were assessed via tensile testing, Charpy V-notch impact tests at −20 °C and Vickers hardness measurements. Microstructural evolution was characterized through optical microscopy (OM), scanning electron microscopy (SEM) and electron backscatter diffraction (EBSD). Key findings reveal that increasing the Cu content from 0.13 wt.% to 0.34 wt.% reduces the volume percentage of acicular ferrite (AF) in the weld metal by approximately 20%, accompanied by a significant decline in cryogenic toughness, with the average impact energy decreasing from 221.08 J to 151.59 J. Mechanistic analysis demonstrates that the trace increase in the Cu element. The phase transition temperature and inclusions is not significant but can refine the prior austenite grain size of the weld, so that the total surface area of the grain boundary increases, and the surface area of the inclusions within the grain is relatively small, resulting in the nucleation of acicular ferrite within the grain being weak. This microstructural transition lowers the critical crack size and diminishes the density for high-angle grain boundaries (HAGBs > 45°), which weakens crack deflection capability. Consequently, the crack propagation angle decreases from 54.73° to 45°, substantially reducing the energy required for stable crack growth and deteriorating low-temperature toughness.

## 1. Introduction

In recent years, the rising global demand for petroleum and natural gas has necessitated pipelines with substantially greater capacity, larger diameters and superior mechanical strength and toughness [1,2,3]. X80 pipeline steel demonstrates superior strength–toughness properties and excellent weld performance. Consequently, it has always been the favored material for long-distance pipelines [4]. However, petroleum and natural gas reserves are generally dispersed in highly cold areas, and the ambient temperature in winter is usually lower than −10 °C, which presents a major challenge to the cryogenic toughness of X80 pipeline steel, especially that of the welds in their weak areas [5,6,7]. As one of the most important elements determining the performance of the weld, the selection and optimization of welding materials are of vital importance. At present, the mainstream welding materials are classified into welding rod, gas-shielded solid wire, submerged arc welding wire, flux-cored wire, flux, etc. [8]. Among them, gas-shielded solid wires have been widely used in pipeline steel welding due to their advantages, such as high production efficiency, strong process adaptability and low welding cost [9]. Therefore, to fulfill the low-temperature toughness requirement of the weld area, it is necessary to conduct research and optimization of the alloy composition of gas-shielded solid wire.

The effects of alloying elements on weld zone microstructure and mechanical capacities in X80 pipeline steel have always been a research hotspot for scholars at home and abroad. Dong et al. studied the effects of Mn, Ni and Mo contents on the microstructure transformation and cryogenic toughness of K65 pipeline steel welds by changing the alloying elements in submerged arc welding wires. The authors believed that the increase in Mn and Ni contents could effectively promote the nucleation and growth of acicular ferrite in the weld metal. When the Mo content in the weld exceeded 0.2%, it could significantly enhance the fraction of AF in the microstructure, allowing the weld metal to achieve outstanding low-temperature toughness [10]. Chu et al. investigated the effects of different submerged arc welding wire compositions on the weld microstructure and mechanical capacities of X80 pipeline steel. The authors found that low alloy contents of C, Ni, Cr and Mo were conducive to the production of acicular ferrite, while high alloy contents of C, Ni, Cr and Mo were conducive to the production of lath bainite. Moreover, welds with low alloy contents of C, Ni, Cr and Mo had better impact energy [11]. To summarize, the current research on welding materials for X80 pipeline steel primarily focuses on submerged arc welding wires containing strengthening and toughening alloy elements (e.g., Mn, Ni, Mo and Cr). In contrast, few studies have investigated the influence of Cu in gas-shielded solid wires on the microstructure and mechanical capacities of X80 pipeline steel welds, although its effects have been extensively studied in other welding fields. Avazkonandeh et al. studied the influence of Cu content on the weld metal of DIN ST52 and found that the proportion of acicular ferrite in the microstructure rose with the rise in Cu content, but the increase in Cu content had a small influence on the grain size of acicular ferrite. The yield strength of the weld rose with the rise in Cu content, and the average impact absorption energy at −40 °C decreased with the rise in Cu content [12]. Evans et al. investigated the effect of Cu content (0.04–1.4 wt.%) on the weld microstructure of C-Mn steel. They observed that as the Cu content increased, the acicular ferrite content decreased; meanwhile, when the proeutectoid ferrite (PF) and ferrite side plate (FSP) contents rose, the weld microstructure became more refined [13]. Li et al. demonstrated that adding Cu greatly improves the mechanical properties of maraging steel weld joints. As the Cu content increased, residual austenite decreased at the grain boundaries while increasing within the matrix, resulting in increased toughness [14]. However, previous studies indicate that the influence of Cu content variations on the weld properties differs considerably across steel types. Furthermore, research on the effects of Cu in gas-shielded solid wires on X80 pipeline steel welds remains limited.

In this study, gas-shielded solid wire containing Cu was used to weld X80 pipeline steel. The influences of Cu on the microstructure and mechanical capacities of X80 pipeline steel weld was analyzed utilizing techniques such as OM, SEM and EBSD. The influences of Cu on grain boundaries, critical crack size and crack propagation angle in the weld microstructure were systematically investigated. This will help understand the influence of Cu on the impact toughness of X80 pipeline steel welds and promote the optimization of the composition system of gas-shielded solid wire for X80 pipeline steel.

## 2. Experimental Materials and Methods

The base material was X80 pipeline steel. Two self-designed gas-shielded solid wires served as the test materials. The welding method is multi-layer and multi-pass welding. Both sides of the bevel are sanded clean with sandpaper until the metallic luster is exposed. Then, the surface dirt is cleaned with acetone alcohol solution, and the surface of the base material for welding is surface-welded to prevent the alloying elements of the base metal from entering the weld. Table 1 shows the main alloying compositions of two types of welds. The welds with 0.13%Cu content and 0.34%Cu content are named 1# and 2#, respectively.

**Figure 1:**
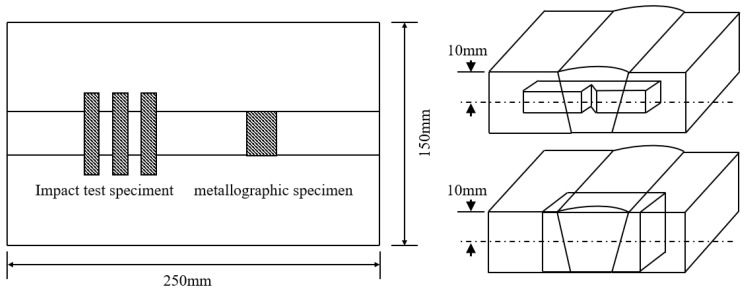
Sampling positions of impact test samples and metallographic samples.

The Charpy impact specimens with dimensions of 55 mm × 10 mm × 10 mm were cut along the vertical direction of the weld. The sampling position is shown in Figure 1. In the center of the Charpy impact specimen, a 2 mm deep V-notch with a 45° angle and 0.25 mm radius of curvature was machined. The Charpy impact test was conducted in accordance with the GB/T19748-2019”Metallic materials-Charpy V-notch pendulum impact test-Instrumented test method” [15] standard, with a test temperature of −20 °C. Tensile specimens with a size of Φ3 mm were processed along the welding direction. The room temperature tensile test was performed using Instron-5958 electronic universal testing equipment with a tensile rate of 1 mm/min, in accordance with GB/T·228-2021 specifications [16]. The tensile and impact test results were taken as the average of the three test values. Ten spots were chosen from various positions in the center of the weld. A hardness test was performed using an FM300 micro-Vickers hardness tester. The load was set at 1 Kg, and the time was 15 s. The microhardness result was calculated by removing the highest and lowest values and taking the average.

A metallographic sample with a size of 30 mm × 20 mm × 20 mm was taken from the weld. The sampling position is shown in Figure 1. After sandpaper grinding and metallographic polishing of the metallographic sample, it was corroded with a 4% nitric acid ethanol solution for 12 s, cleaned and dried. The metallographic microstructure of the weld was viewed using an optical microscope. The microstructure of the weld was further observed and analyzed by using thermal field emission scanning electron microscopy (FEI Quanta650 FEG, Field Electron and Ion Company, CA, USA). The impact fractures were ultrasonically cleaned with anhydrous ethanol to remove surface contaminants, and the fracture morphology and crack propagation path were observed via SEM. Weld inclusions were observed using SEM with a magnification of 2000×. Ten fields of view were randomly chosen, and the quantity, size and characteristics of the inclusions were statistically analyzed using Image-J image V1.53 analysis software. The composition of the inclusions was analyzed using SEM companion (energy-dispersive spectroscopy, EDS). The specimens of the weld were electrolytically polished using a 10% perchloric acid ethanol solution. The electrolytically polished weld specimens were analyzed via EBSD using a scanning electron microscope equipped with electron backscatter diffraction. The scanning step size was 0.2 µm, and the area was 100 µm × 100 µm. The acquired EBSD images were processed using KCL-Channel5 V5.12.74.0 software to calculate the proportion of high-angle grain boundaries, grain size and to analyze grain orientation.

## 3. Experimental Results and Analysis

### 3.1. Mechanical Properties

**Figure 2:**
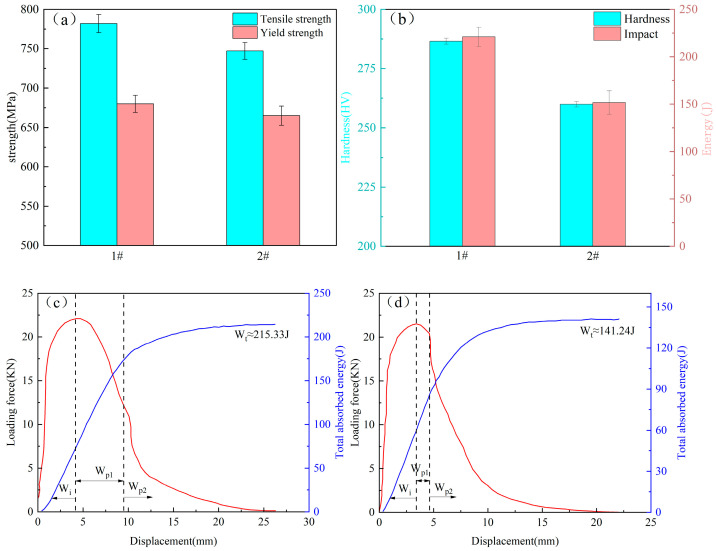
Mechanical properties of welds with different Cu contents: (**a**) Tensile properties; (**b**) Hardness and impact work; (**c**) Loading force–displacement curve of 1# weld; (**d**) Loading force–displacement curve of 2# weld.

Figure 2 shows the mechanical properties of the two welds. Figure 2a shows the tensile test results. 1# weld has better tensile strength (782 MPa) and yield strength (680 MPa) compared to 2# weld, while the yield ratio of 1# weld is smaller than that of 2# weld. Studies have shown that high-strength steels experience complex loading conditions during service; therefore, reducing the yield ratio of steel structures appropriately can enhance structural safety [17]. Figure 2b shows the microhardness and −20 °C impact performance test results. The microhardness of 1# weld is significantly higher than that of 2# weld. Concerning the impact property, the impact energy of the two welds is more than 150 J. Among them, 1# weld shows more excellent impact performance, with its impact energy reaching 221 J, higher than that of 2# weld at 151 J by 46.4%.

Oscillation impact tests were adopted for further study and analysis of the toughness of weld specimens. Figure 2c,d show quintessential loading force–displacement curved lines on the basis of the Charpy impact test for 1# and 2# welds. It is generally recognized that the entire impact absorption energy of a material may be decomposed into two stages: crack initiation energy (W_i_) and crack propagation energy (W_P_). Among them, W_i_ corresponds to the energy accumulated before the load peaks, and W_P_ corresponds to the energy accumulated after the load peaks [18,19]. The two figures reveal that although the difference in peak-load forces between the two welds is insignificant, the crack initiation energy of 1# weld (72.64 J) is higher than that of 2# weld (58.93 J), which indicates that 2# weld is more sensitive to crack initiation and is more prone to cracking. In addition, 1# weld has a conspicuous stable crack propagation stage (W_P1_), and the final crack tearing absorbed energy (W_P2_) decreases slowly. 2# weld’s W_P1_ is significantly smaller than that of 1# weld, and W_P2_ decreases rapidly. This indicates that the crack expansion process of 2# weld is faster, which is unfavorable to the buildup of total absorbed energy.

Table 2 sets out some typical curve numerics and impact toughness numerics for the two welds, where F_y_ is the general yield force; F_m_ is the maximum force; and F_a_ is the fracture arrest force. Figure 2c,d reveal that both welds exhibit different crack propagation behaviors, and the ability of the material to resist crack extension can be effectively characterized by the F_a_/F_m_ ratio; the larger the ratio, the better the fracture prevention performance [20]. The value of Fa/Fm for 1# weld is 0.306, which is higher than the value of 0.188 for 2# weld. These results indicate that 1# weld has a higher toughness than 2# weld.

### 3.2. Weld Microstructure

**Figure 3:**
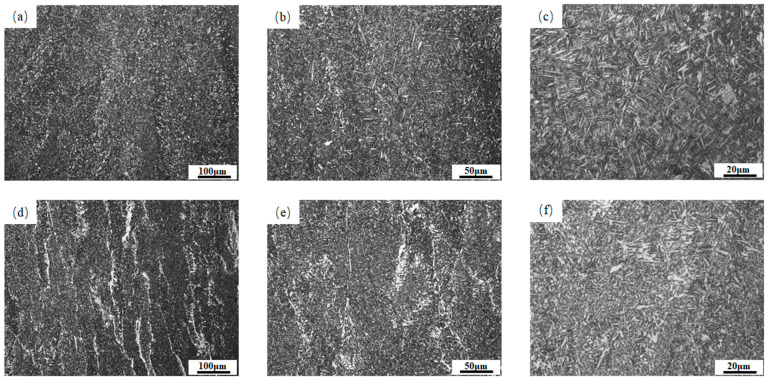
The microstructure of the two welded metals: (**a**) 1#—100×; (**b**) 1#—200×; (**c**) 1#—500×; (**d**) 2#—100×; (**e**) 2#—200×; (**f**) 2#—500×.

Figure 3 shows the metallographic microstructures of the central area of the cosmetic welding of the two welds. Both weld microstructures are predominantly composed of acicular ferrite, with minor white regions distributed within the matrix. Figure 3a reveals that the white regions in 1# weld are more dispersed, and no dendritic structure is formed. Upon magnification, these regions are primarily identified as proeutectoid ferrite. Figure 3d reveals that compared to 1# weld, the white regions in 2# weld are more concentrated and exhibit distinct dendritic morphology. Upon magnification of 2# weld, white regions with massive proeutectoid ferrite content can clearly be observed, as well as a tooth-shaped parallel arrangement to one side of the crystal growth of the ferrite side plate. Image-J software was used to analyze 10 metallographic images at 200× magnification to quantify the proportion of white regions in 1# weld and 2# weld. The results showed that 1# weld contained approximately 10% white regions compared to 30% for 2# weld.

**Figure 4:**
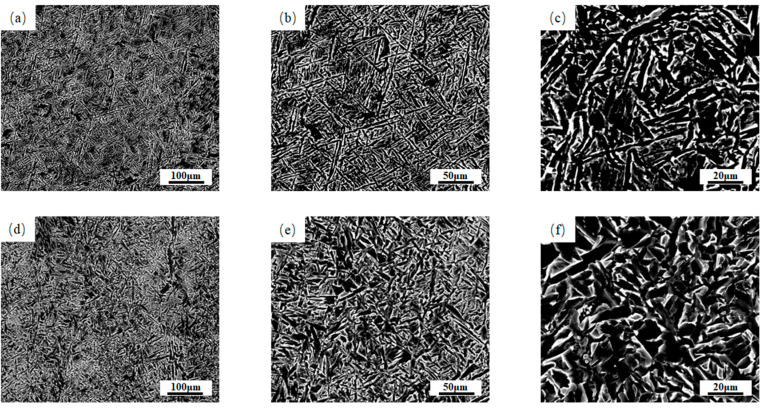
SEM images of welds with different copper contents at different magnifications: (**a**–**c**) SEM images of 1# weld at 1000×, 2000× and 5000×, respectively; (**d**–**f**) SEM images of 2# weld at 1000×, 2000× and 5000×, respectively.

Figure 4 shows SEM images of the two types of weld microstructures. The microstructures of the two types of welds were characterized in detail via scanning electron microscopy. It can be observed that 1# weld acicular ferrite mainly takes the form of tightly arranged “interlocking”, with a slenderer grain size; the “interlocking” morphology of acicular ferrite in 2# weld is less pronounced compared to that in 1# weld. This is mainly attributed to the fact that some ferrite side plates are scattered in the acicular ferrite in 2# weld, and these ferrite side plates are spaced between the acicular ferrites, hindering acicular ferrite interconnections. Moreover, the grain length of the acicular ferrite in 2# weld is shorter than that in 1# weld. This is not conducive to the formation of the “interlocking” structure. Related studies have shown that the “interlocking” structure of acicular ferrite is high-angle grain boundaries, which may effectively impede crack propagation and thus enhance the toughness of the material [21].

**Figure 5:**
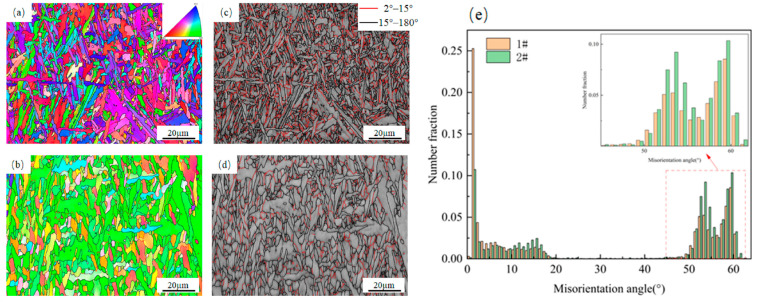
EBSD results of two welds with different copper contents: (**a**,**c**) Inverse pole figure and grain boundary figure of the microstructure of 1# weld; (**b**,**d**) Inverse pole figure and grain boundary figure of the microstructure of 2# weld; (**e**) The proportion of grain boundaries at high and low angles of the two welds to the misorientation angle.

The EBSD technique enables more intuitive observation of the microstructures. To study the microstructural differences between the two welds, EBSD analysis was conducted on both weld samples. Figure 5a,b show the grain orientations of the two types of welds with varying Cu contents, and the triangles in the upper right corner represent the grain orientations that correspond to different colors. The microstructure of the two types of welds is a mixture of acicular ferrite and proeutectoid ferrite, but the proportions and sizes are different. It is evident that the proeutectoid ferrite content in 2# weld is higher, while the acicular ferrite in 1# weld exhibits a more uniform distribution and slender morphology compared to 2# weld. Moreover, in the EBSD image, the color difference between the adjacent grains of #1 weld is obvious, which indicates that the adjacent acicular ferrite grains of #1 weld have different crystal orientations. This difference in orientation contributes to the formation of a uniform acicular ferrite “interlocking” structure.

According to the orientation angle difference between the grains on either side of the grain boundary, grain boundaries with angles > 15° are defined as high-angle grain boundaries (HAGBs), while those with angles < 15° are low-angle grain boundaries (LAGBs). According to research, high-angle grain boundaries can effectively improve fracture resistance by hindering crack propagation [22,23,24]. In Figure 5c,d, the black lines indicate HAGBs with a misorientation angle of >15°, and the red lines indicate LAGBs with a misorientation angle of <15°. Figure 5e shows the variation law of the proportion of grain boundaries at high and low angles to the misorientation angle. The results show that the percentage of HAGBs is 71% for 1# weld and 77% for 2# weld.

**Figure 6:**
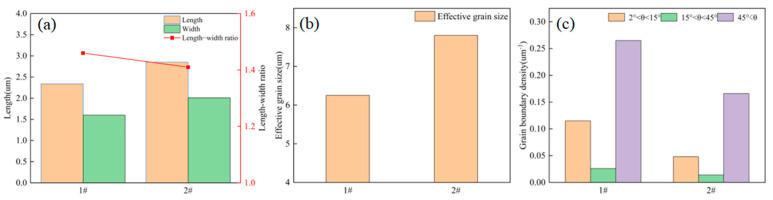
Grain information analysis of two weld metals: (**a**) Aspect ratio diagram; (**b**) Effective grain size diagram; (**c**) Grain boundary density diagram.

Although the low-temperature toughness of 1# weld is superior to that of 2# weld, its percentage of HAGBs is lower than that of 2# weld—a phenomenon, which cannot explain the fracture toughness results. Therefore, from each of the two welds, 1000 grains were selected. Their aspect ratios and effective grain sizes were measured, and the grain boundary density unit areas were computed. These measurements were used to analyze the micromechanisms of fracture behavior. Figure 6a shows the statistical results of the aspect ratios of the grains of the two types of welds. The results show that the grain width of 1# weld is significantly smaller than that of 2# weld, but the aspect ratio of the two is similar. This indicates that the grains of 1# weld maintain a similar slender shape as their size decreases. Figure 6b shows the effective grain size statistics of the two welds, showing that the grain size of 1# weld is significantly smaller than that of 2# weld. Since the orientation difference distribution map cannot compare the proportion of high-angle grain boundaries between the two welds, grain boundary densities of 2–15°, 15–45° and over 45° in the two types of welds are statistically analyzed. Figure 6c shows the statistical results of grain boundary density. These data reveal distinct grain boundary densities per unit area across three ranges (2–15°, 15–45°, >45°) in both welds. Specifically, in the >45° range, the grain boundary density in 1# weld exceeds that in 2# weld by 37.5%. With the reduction in effective grain size and enhancement in the grain boundary density over 45°, the high-angle grain boundaries per unit area are obviously increased, which enhances the ability to hinder the dislocation slip and increases the strength and low-temperature toughness of 1# weld [25].

## 4. Discussion

### 4.1. The Influence of Cu on the Weld Microstructure

#### 4.1.1. The Influence of Copper on the Phase Transformation Temperature

Based on the above microstructure observations, the primary difference between the two kinds of welds lies in the content of acicular ferrite. In the welding test, except for the percentages of alloying elements in the gas-shielded solid wire being different, the rest of the welding conditions are basically the same. Therefore, in this paper, alloying elements are the main factors in determining the microstructure of the weld metal. As shown in Table 1, except for the Cu content in the weld being significantly different, the content of the other elements basically remains the same. Thus, the discussion will focus specifically on the effect of Cu on acicular ferrite nucleation. Related studies have shown that alloy composition, inclusions and prior austenite grain size are the main factors affecting acicular ferrite nucleation [26,27,28].

**Figure 7:**
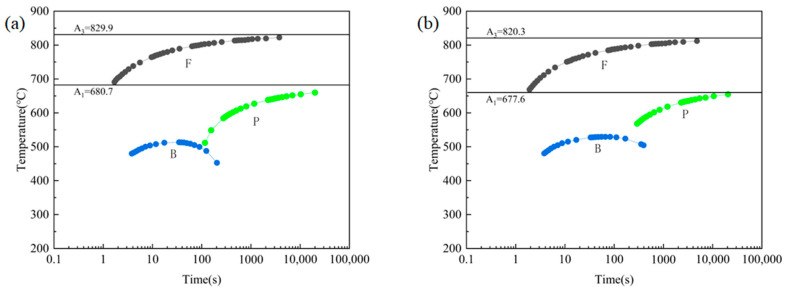
JmatPro-simulated weld CCT results: (**a**) 1#; (**b**) 2#.

Cu is an austenite-stabilizing element that can lower the phase transformation temperature of the weld metal, promote the rightward shift of the continuous cooling transformation (CCT) curve and contribute to acicular ferrite formation [29,30]. This is contrary to the results of microstructure observation; therefore, based on the alloying elements in Table 1, simulations were carried out using JmatPro 7.0.0 software to obtain their CCT curves. As shown in Figure 7, the A_3_ temperature of the two welds was 829.9 °C and 820.3 °C, respectively, while the A_1_ temperature was 680.7 °C and 677.6 °C, respectively. The A3 and A1 temperatures of the weld with a Cu content of 0.34% showed decreases of 9.6 °C and 3.1 °C, respectively, compared to those of the weld with a Cu content of 0.13%. The calculation results indicated that phase transition temperature exerted little effect on either weld because the Cu content was below 0.4%.

#### 4.1.2. The Influence of Copper on the Characteristics of Inclusions

**Figure 8:**
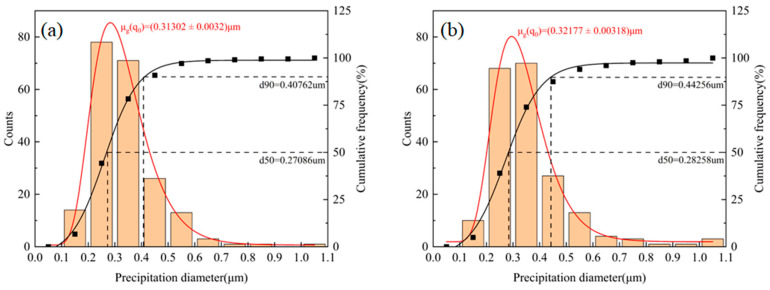
Statistics on the size distribution of inclusions per unit area of the welds: (**a**) 1#; (**b**) 2# (In the figure, μg (q_0_) denotes the average size; d90 denotes the maximum particle size for the first 90% of inclusions; d50 denotes the maximum particle size for the first 50% of inclusions).

The number and size distribution of the inclusions in the 10 SEM images were calculated using the Image-J image analysis software. As shown in Figure 8, it can be observed that the size and the number of inclusions per unit area in the two kinds of welds are basically the same, and the size distribution of the inclusions is within 1 μm, most of which are distributed between 0.2 and 0.4 μm, with small-sized inclusions dominating. The number density of the inclusions in the two weld metals was calculated as 9.123 × 10^3^ mm^−2^ and 8.772 × 10^3^ mm^−2^, with average sizes of 0.313 μm and 0.322 μm, respectively. The cumulative size distributions at 50% and 90% were similar for both welds. The calculation results show that the quantity and size of inclusions per unit area in the two weld metals are basically the same. The results show that the increase in the Cu element has almost no effect on the number and size of inclusions.

Five inclusions were randomly selected from the two weld metals, and the compositions were analyzed using EDS. Table 3 shows the test results. The inclusion composition consisted of Ti, Mn, S, O and Si. A comparison of the two kinds of welds showed that the inclusion composition remained nearly constant with increasing Cu content, indicating that Cu does not affect the chemical properties of inclusions, suggesting that Cu does not influence the nucleation of AF by altering the chemical properties of inclusions in the welds.

#### 4.1.3. The Influence of Copper on the Prior Austenite Grain Size

The austenite-to-ferrite transformation requires a surface to form the new phase; therefore, the type of ferrite formed on the surface of grain boundaries depends on the total surface area of the intra-metallic grain boundaries and within-grain inclusion particles. The nucleation ability and volume fraction of intra-granular acicular ferrite increase with the ratio of the total surface area of inclusion particles to the total surface area of grain boundaries [31]. Therefore, in the case of constant total surface area of inclusion particles, the increase in the total surface area at grain boundaries will reduce the nucleation ability and volume fraction of intra-crystalline acicular ferrite. Cu is an austenite-stabilizing element; it can increase the volume fraction for austenite, and its drag effects can effectively refine the dimension of the prior austenite grains [13,32]. Therefore, when the Cu element content of 2# weld is 0.34%, its prior austenite grain size is finer compared to that of 1# weld with a Cu element content of 0.13%, which makes the total surface area of the grain boundary of 2# weld relatively larger, resulting in the volume fraction for acicular ferrite of 2# weld metal being lower than that of 1# weld metal.

### 4.2. The Influence of Weld Microstructure on Critical Crack Size

The critical crack size is the smallest crack length that will initiate instability extension (fracture failure) of a material or structure when the crack length in the material or structure reaches a specific critical value [33,34]. Therefore, the larger the critical crack size, the more resistant the material is to fracture. This relationship means that critical crack size calculations can effectively predict material toughness. Therefore, it is necessary to calculate the critical crack size. The dynamic yield stress (*σ_yd_*) can be determined using Equation (1) [35]:

(1) $$\sigma_{yd}=467F_{y}/B$$

In Equation (1), B represents the thickness of the Charpy impact specimen in mm, and *F_y_* is the general yield load in kN. The strain rate significantly affects the yield stress of a material, and under high-strain-rate conditions, the dynamic yield stress of a material is much higher than the static yield stress. Therefore, the critical stress (*σ_c_*) can be confirmed using the following Equation (2) [35], where the stress enhancement factor Cf for the Charpy notched specimen is taken as 2.24 [36].

(2) $$\sigma_{c}=c_{f}\sigma_{yd}$$

For microporous cracks, which are elliptical in shape, the critical microcrack size is calculated according to Griffith’s formula Equation (3) [36,37]:

(3) $$\sigma_{c}={\left(\frac{\pi E\gamma_{p}}{\left(1-v^{2}\right)d}\right)}^{1/2}$$

Here, *E* is Young’s modulus; *γ_p_* is the effective surface energy of the microcrack; *v* represents Poisson’s ratio; and *d* represents the length of the critical crack size. According to relevant research, an *E* of 210 GPa, *γ_p_* of 14 J/m^2^ and *v* of 0.3 are adopted as the values for the above formula [38]. The calculated results show that the critical crack size of the 1# weld specimen is about 4 μm, while the critical crack size of the 2# weld specimen is approximately 3.6 μm. Since larger critical crack size indicates better material toughness, according to the calculation results of the above formula, the toughness of 1# weld is superior to that of 2# weld.

### 4.3. The Influence of Weld Microstructure on Crack Expansion Angle

**Figure 9:**
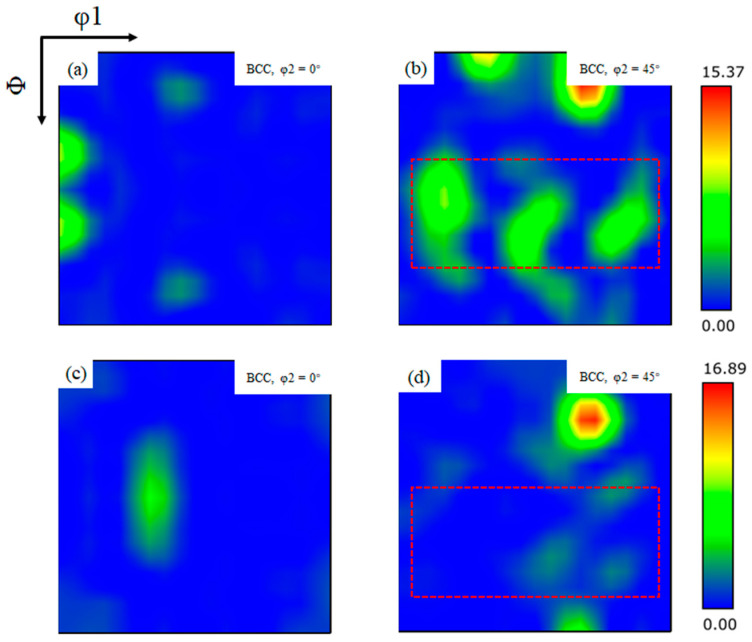
ODF sections with different Euler angles φ2: (**a**,**b**) ODF section of 1# weld φ2 = 0° and φ2 = 45°; (**c**,**d**) ODF section of 2# weld φ2 = 0° and φ2 = 45°.

For ferrite with a cubic crystal system BCC structure, the important grain orientation is often determined by analyzing the position of the characteristic orientation in the φ2 = 0° and φ2 = 45° cross-sections [39]. Figure 9 shows the grain ODF figures of the two welds. It can be observed that the grain orientations of the two welds are not exactly the same. Through the orientation distribution function proposed by Bunge H J, it can be calculated that the ferrite grain orientation of 1# weld at φ2 = 0° is mainly distributed in the vicinity of {110} <001> and that the ferrite grain orientation at φ2 = 45° is mainly distributed in the vicinity of {111} <110> and {100} <001>. 2# weld at φ2 = 0° ferrite grain orientation is mainly distributed in the vicinity of {110} <112>, while at φ2 = 45° ferrite grain orientation, it is mainly distributed in the vicinity of {100} <001>.

According to Schimid’s law, the smaller the angle between the crystal plane and the direction where cracks can most easily propagate, the more likely it is that rotation will occur during crack propagation, resulting in easier crack propagation [40]. In the cubic crystal BCC system, the angle between the {100} crystal plane and the {110} crystal plane is 45°; the angle between the {100} crystal plane and the {111} crystal plane is 54.73°.. In the previous section, it can be observed that the ferrite in 2# weld is mainly distributed in the vicinity of {110}<112> and {100}<001>; the angles between them and the {100} most extended crystal surface of cracks in the cubic crystal BCC system are 0° and 45°, respectively. Meanwhile, the ferrite in 1# weld is distributed in the vicinity of {110}<001>, {111}<110> and {100}<001>; the angles between them and the {100} crystal surface are 0°, 45° and 54.73°, respectively. When the percentage of the Cu element in the weld is 0.13%, the orientation of ferrite grains and the angle between the most easily extended surfaces of cracks tend to increase, resulting in an increase in the resistance to crack extension of 1# weld, which improves the toughness of the weld.

### 4.4. Fracture Morphology Analysis

#### 4.4.1. Fracture Morphology

**Figure 10:**
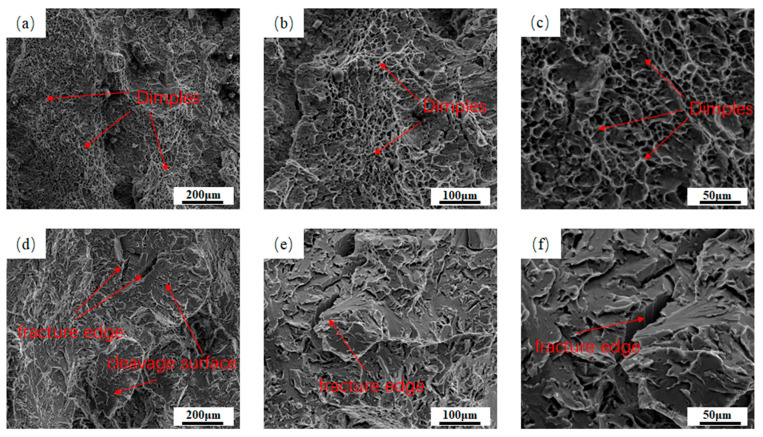
SEM images of the fracture surface morphology in the impact specimen at −20 °C: (**a**–**c**) SEM images of 1# weld impact fracture at 500×, 1000× and 2000×; (**d**–**f**) SEM images of 2# weld impact fracture at 500×, 1000× and 2000×.

Figure 10 shows the scanning electron microscopy images of the impact fractures of the two kinds of welds at −20 °C. Figure 10a–c show the fracture morphology of 1# weld; it can be seen that its fracture mainly consists of large and deep dimples, which is a typical toughness fracture morphology. This is conducive to prolonging the stable crack extension stage (Figure 2c), increasing the absorbed energy of crack extension (Table 2) and helping to improve the toughness of the material [41]. Figure 10d–f show the fracture morphology in 2# weld; it can be seen that its fracture presents typical quasi-cleavage fracture characteristics, which are mainly manifested as an obvious cleavage surface, and there are deep and wide cracks on the cleavage surface. Obviously, the low-temperature impact toughness in 1# weld is superior to that in 2# weld, which is consistent with the impact energy data shown in Table 2.

#### 4.4.2. Crack Propagation Path

**Figure 11:**
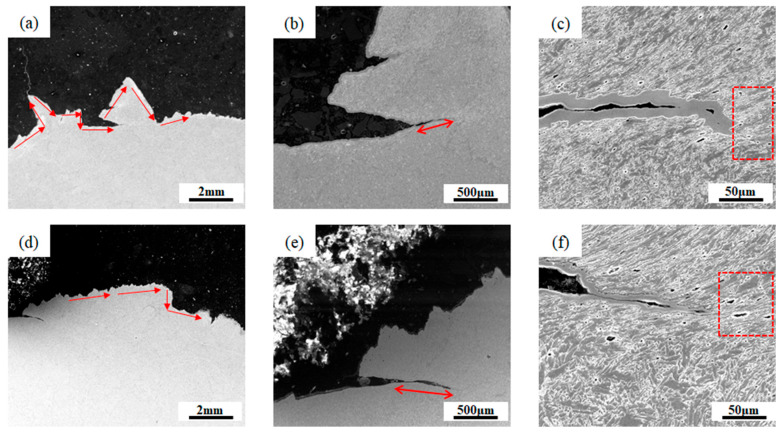
SEM image of the cracking trend of 1# and 2# weld metals: (**a**) The main crack path of 1# weld metal; (**b**,**c**) The partial enlarged view of (**a**); (**d**) The main crack path of 2# weld metal; (**e**,**f**) The partial enlarged view of (**d**).

Figure 11 shows the SEM images of the fracture morphology of the longitudinal sections of the two kinds of welds. Figure 11a,d show the crack extension paths of the two welds, respectively. It can be found that the crack propagation path in 1# weld is more tortuous, and the total length of the crack is longer, while the crack extension path in 2# weld is straighter and has fewer twists and turns. Figure 11b,e show an enlarged view of the secondary cracks. It can be seen that the length of the secondary crack in 1# weld is shorter, and the width is narrower than that in 2# weld. The magnified view in Figure 11c,f reveals numerous micropores distributed around the secondary crack. It is noteworthy that the number and size of micropores in 1# weld are significantly smaller than those in 2# weld. This phenomenon corresponds well with the results of the critical microcrack size calculated above: since the critical microcrack size of 2# weld is smaller, it is easier for micropores to form, thus providing more nucleation sites for the crack [42]. The expansion behavior of the secondary crack shows that the crack will deflect or stop expanding when it encounters AF again, which indicates that AF is beneficial for increasing the crack expansion resistance and thus improving the impact toughness of the material.

## 5. Conclusions

This paper systematically investigates the influence of adding different Cu contents to gas-shielded solid wires on the mechanical properties and microstructure in X80 pipeline steel welds. The main findings are as follows:

1. When the Cu content in the weld increases from 0.13% to 0.34%, the low-temperature impact toughness declines from 221.08 J down to 151.59 J, representing a 31.4% reduction in impact toughness.
2. When the Cu content in the weld increases, the content of acicular ferrite in the weld tissue decreases by about 20%, and the effective grain size of the weld increases by about 25%.
3. The reduction in acicular ferrite content in the weld leads to a decrease in the critical crack size and high-angle grain boundary density, which reduces the crack extension angle from 54.73° to 45°. The steady-state crack propagation energy is significantly reduced, leading to a smoother crack propagation path and ultimately diminishing the low-temperature toughness.

## Figures and Tables

**Table 1:** Alloying compositions of weld metal (wt.%).

No.	C	Si	P	S	Ti	Cr	Mn	Ni	Cu	Mo
1#	0.091	0.72	0.007	0.005	0.045	0.053	1.38	0.81	0.13	0.034
2#	0.095	0.65	0.005	0.008	0.042	0.057	1.41	0.80	0.34	0.052

**Table 2:** Charpy instrument impact test results for 1#weld and 2# weld.

No.	F_y_ (kN)	F_m_ (kN)	F_a_ (kN)	W_i_ (J)	W_p_ (J)	Cryogenic Impact Toughness (J)
1#	15.37	22.46	6.88	72.64	140.96	221.08 ± 10.12
2#	16.02	21.54	4.05	58.93	81.29	151.59 ± 12.38

F_y_: general yield force, F_m_: maximum force, F_a_: fracture arrest force, W_i_: crack initiation energy, W_p_: crack propagation energy. The Cryogenic Impact Toughness result is the average of the entire absorption energy of the three specimens.

**Table 3:** Composition of weld metal inclusions with different Cu content (wt.%).

NO.	Ti	Mn	S	O	Si	Fe
1#	16.6	6.6	0.4	22.1	0.8	53.5
2#	18	9	0.8	20	0.4	51.8

## Data Availability

The original contributions presented in the study are included in the article, further inquiries can be directed to the corresponding author.
